# Use of Ionic Liquids in Chitin Biorefinery: A Systematic Review

**DOI:** 10.3389/fbioe.2020.00011

**Published:** 2020-01-31

**Authors:** Julia L. Shamshina, Paula Berton

**Affiliations:** ^1^Mari Signum, Richmond, VA, United States; ^2^Chemical and Petroleum Engineering Department, University of Calgary, Calgary, AB, Canada

**Keywords:** biorefinery, chitin, biomass, ionic liquids, circular economy, biomass valorization

## Abstract

Lignocellulosic biomass biorefinery is the most extensively investigated biorefinery model. At the same time, chitin, structurally similar to cellulose and the second most abundant polymer on Earth, represents a unique chemical structure that allows the direct manufacture of nitrogen-containing building blocks and intermediates, a goal not accomplishable using lignocellulosic biomass. However, the recovery, dissolution, and treatment of chitin was fairly challenging until the polymer's easy dissolution in ionic liquids (salts that are liquid at room temperature) was discovered. In this systematic review, we highlight recent developments in the processing of chitin, with a particular emphasis placed on methods conducted with the help of ionic liquids used as solvents, co-solvents, or catalysts. Such use of ionic liquids in the field of chemical transformations of chitin not only allows for shorter times and less harsh reaction conditions, but also results in different outcomes and higher product yields when compared with reactions conducted in “traditional” manner. Valorization of biomass in general, and chitin in particular, is a key enabling strategy of the circular economy, due to the importance of the sustainable production of biomass-based goods and chemicals and full chain resource efficiency. Economics is driven by the production of high-value chemicals or chemical intermediates from various biomasses, and chitinous biomass is a valuable potential resource. A fundamental “paradigm shift” will radically change the balance of oil-based chemicals to biopolymer-based chemicals, and chitin valorization is a necessary step aimed toward its full market competitiveness and flexibility.

## Biorefinery and Chitin Biopolymer

Today, there is a strong drive toward the circular economy of producing materials and chemicals from sustainable sources (Mitchell, 2018) caused by the devastating quantity of plastics and the limits of oil resources (North and Halden, 2013). In this regard, biorefining is one of the key enabling strategies of the circular economy, with a primary purpose of developing biomass-based products and chemicals rather than oil-based ones. The concept is similar to petrochemical refineries where feed crude oil, initially consisting of several thousand different organic compounds, is first separated into major streams and then chemically converted into pure building blocks for consumer goods (de Jong and Jungmeier, 2015). Similarly, biomass feedstock can be partially or completely separated into main fractions, followed by the deconstruction of the components into valuable materials, chemicals, and biofuels (Stöcker, 2008; Stark, 2011; North and Halden, 2013; Sun et al., 2017). A great example of biorefinery that uses lignocellulosic biomass is the National Renewable Energy Laboratory (NREL), where an Integrated Biorefinery Research Facility (IBRF) was built encompassing a pilot plant on site to “develop, test, evaluate, and demonstrate processes and technologies for the production of bio-based products and fuels (NREL, 2019).”

**Figure 1:**
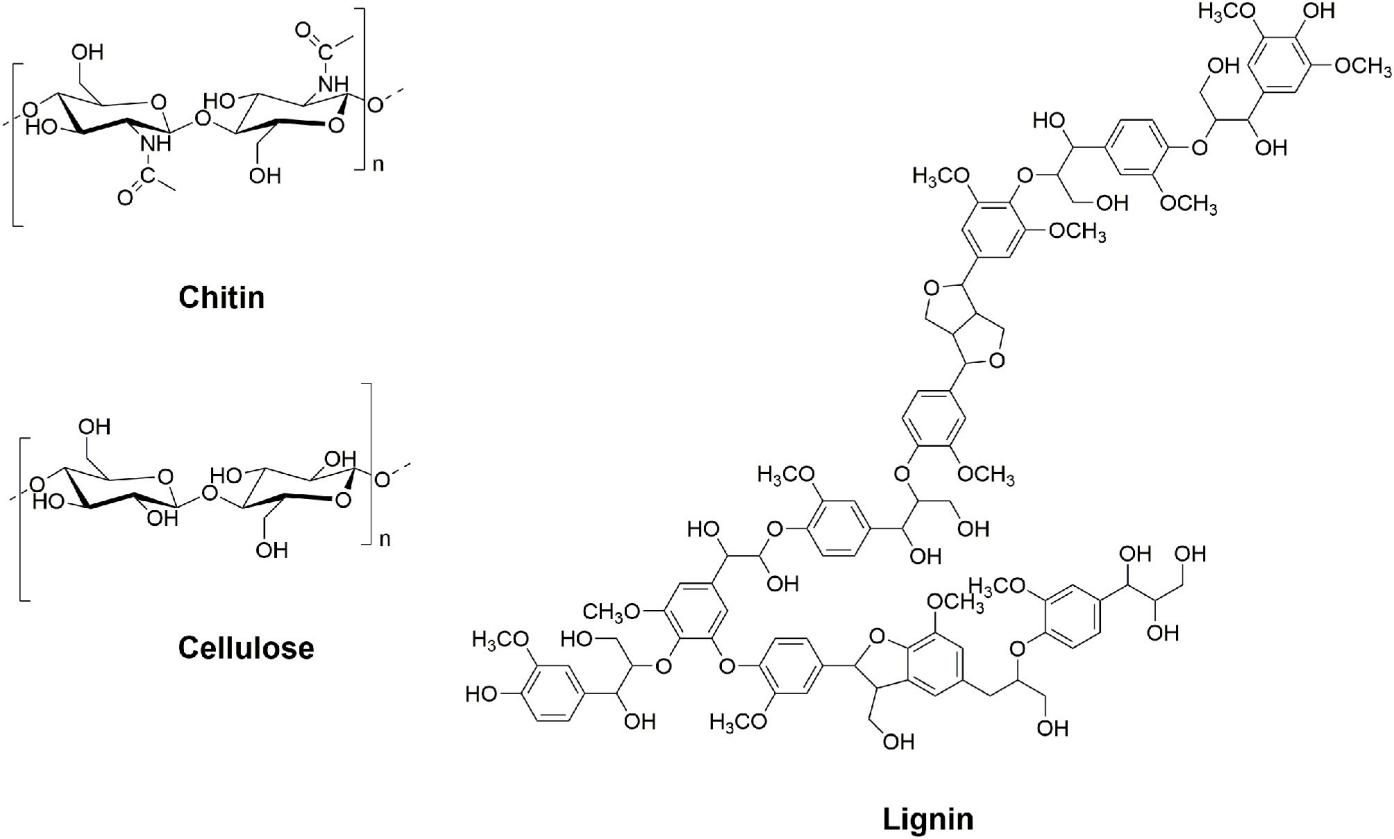
Structure of chitin **(Top Left)**, cellulose **(Bottom Left)**, and lignin **(Right)**.

We believe biopolymers, such as chitin, cellulose, or lignin (Figure 1), are of great interest due to their unique set of properties and characteristics suitable for direct preparation of functional materials in many fields, and we will always strongly advocate for using unmodified polymers as-is, for the preparation of materials directly (Qin et al., 2010; Barber et al., 2013; Shamshina et al., 2014, 2019; Rogers, 2015; Shen et al., 2016; King C. A. et al., 2017; King C. et al., 2017). However, following the practice with oil-based products, these biopolymers can be cut up into building blocks for production of low-volume, but high-value, chemicals. This is especially true for biopolymers such as lignin, with a highly variable composition. Whereas, a lot of research has been conducted on the topic of lignocellulosic biomass transformation (de Jong and Gosselink, 2014), much less research has been done on the treatment of chitin.

Chitin, the second most copious polysaccharide on Earth (after cellulose) (Elieh-Ali-Komi and Hamblin, 2016), is structurally analogous to cellulose except for the acetamide side chain at the C-2 position (Figure 1). It is a linearly assembled 2-(acetylamino)-2-deoxy-D-glucose β-linked polymer (Foster and Webber, 1961), available mostly from shrimp and crab shell waste biomass (15–40%) (Younes and Rinaudo, 2015), where it coexists with proteins (20–40%) and minerals (20–50%) (Rhazi et al., 2000; Ibitoye et al., 2018). Its abundance (*ca*. 0.15 megatons per year) (Roberts, 1992) makes chitin a remarkably valuable potential resource for the production of value-added chemicals. Because chitin has 5–7 wt% of biologically fixed nitrogen (value that depends on its degree of acetylation or DA) (Schoukens, 2009), it is possible to directly produce nitrogen-containing building blocks from this polymer. This is a task which cannot be accomplished using lignocellulosic biomass. Chitin possesses a unique combination of properties and is widely used in preparation of chemicals and materials, mostly for the biotechnology market (Prudden et al., 1970; Peluso, 1994; Jayakumar et al., 2011; Yang, 2011; Vázquez et al., 2013; Wan and Tai, 2013; Shamshina et al., 2018).

**Scheme 1:**
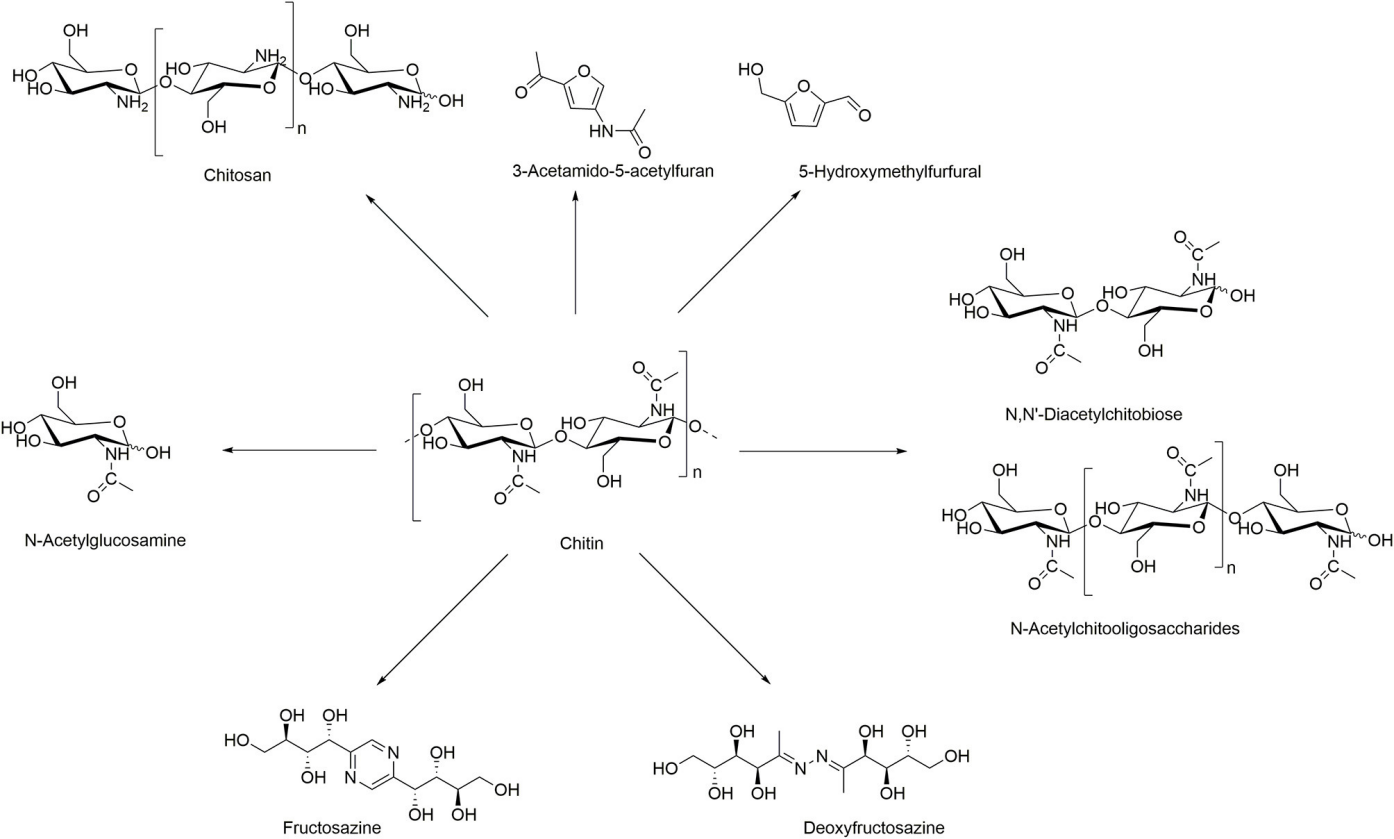
Chitin conversion to high-value chemicals.

Chitin can be transformed into valuable chemicals using transformations shown in Scheme 1, such as the hydrolysis to its monomer *N*-acetylglucosamine (GlcNAc), dimer *N, N*′-diacetylchitobiose, or longer oligomers, where the hydrolysis of the glycosidic linkages is much faster than deacetylation (Einbu and Vårum, 2008). Also, its deacetylation is widely explored to generate chitosan of different DAs, obtained using more or less harsh conditions, depending on the potential applications (Sivashankari and Prabaharan, 2017). Less explored reactions of chitin include formation of furan derivatives such as 5-hydroxymethylfurfural (5-HMF) and nitrogen-containing (*N*-containing) furan derivatives such as 3-acetamido-5-acetylfuran (3A5AF), normally conducted with the use of acidic catalysts and dehydration agents (Omari et al., 2012). Other, less known, chemical transformations include the purification of fructosazine and deoxyfructosazine, normally obtained from glucose under weakly acidic reaction conditions (Tsuchida et al., 1973).

Perhaps one of the main reasons for the limited exploratory work for chitin in comparison with cellulose can be explained by its insolubility in water and most organic solvents (Sieber et al., 2018). In this regard, the field of ionic liquids (ILs, organic salts with melting points below 100°C) (Hayes et al., 2015) offers tremendous potential as a class of solvents suitable for chitin processing. Due to their ability to directly dissolve both biomass (Fort et al., 2007; Kilpeläinen et al., 2007; Sun et al., 2009; V2020 Alternate Feedstock Report, 2019) and purified biopolymers (Phillips et al., 2004; Heinze et al., 2005; Xie et al., 2005, 2006; Zhang et al., 2005; Biswas et al., 2006; Pu et al., 2007; Wu et al., 2008), ILs have opened a door to effectively explore the carbohydrate economy and are widely used for biomass conversion. At the same time, the use of chitin as a sustainable resource assumes employment of more sustainable methods for its processing.

Indeed, the use of ILs for biopolymer processing presents a great advantage. Because ILs are capable of the disruption of chitin's hydrogen bonds, ILs have been shown to act as agents for biopolymer (or biomass) pre-treatment and solvents/co-solvents (Heinze et al., 2005; Biswas et al., 2006; Wu et al., 2008; Sun et al., 2009). Hence, chitin polymer can be pre-treated or even dissolved in the suitable IL prior to its transformation, with its hydrogen bond network being partially or fully destroyed. This provides an amorphous chitin of lower degree of crystallinity and makes the polymer chain “opened” and fully accessible to the reactant(s). ILs are also used as acidic catalysts (e.g., -SO_3_H functionalized ILs, where -SO_3_H is a part of cation or anion demonstrated high catalytic activity compared with non-functionalized ILs) (Sharghi et al., 2018) and as solvents or co-solvents at the same time. Besides, in many areas, reactions conducted using ILs as solvents, catalysts, or reactants have different outcomes and/or product yields when compared with those done in “traditional” manner. Furthermore, reactions utilizing ILs often require less harsh conditions and shorter reaction times (Maier et al., 2017).

Despite of all these achievements, the field of converting chitin into *chemicals* using ILs is still in its infancy. This fact is rather surprising, considering the structural similarity between chitin and cellulose and the significant efforts from industry and research centers (e.g., Joint Bioenergy Institute, JBEI, 2019)^1^ in maximizing the value derived from the lignocellulosic biomass feedstock with the help of ILs. This systematic review will hence focus on the different approaches explored on processing the chitin polymer with the help of ILs for production of sugars, aldehydes, nitrogen-containing compounds, and further chemical transformations.

## Hydrolysis

**Scheme 2:**
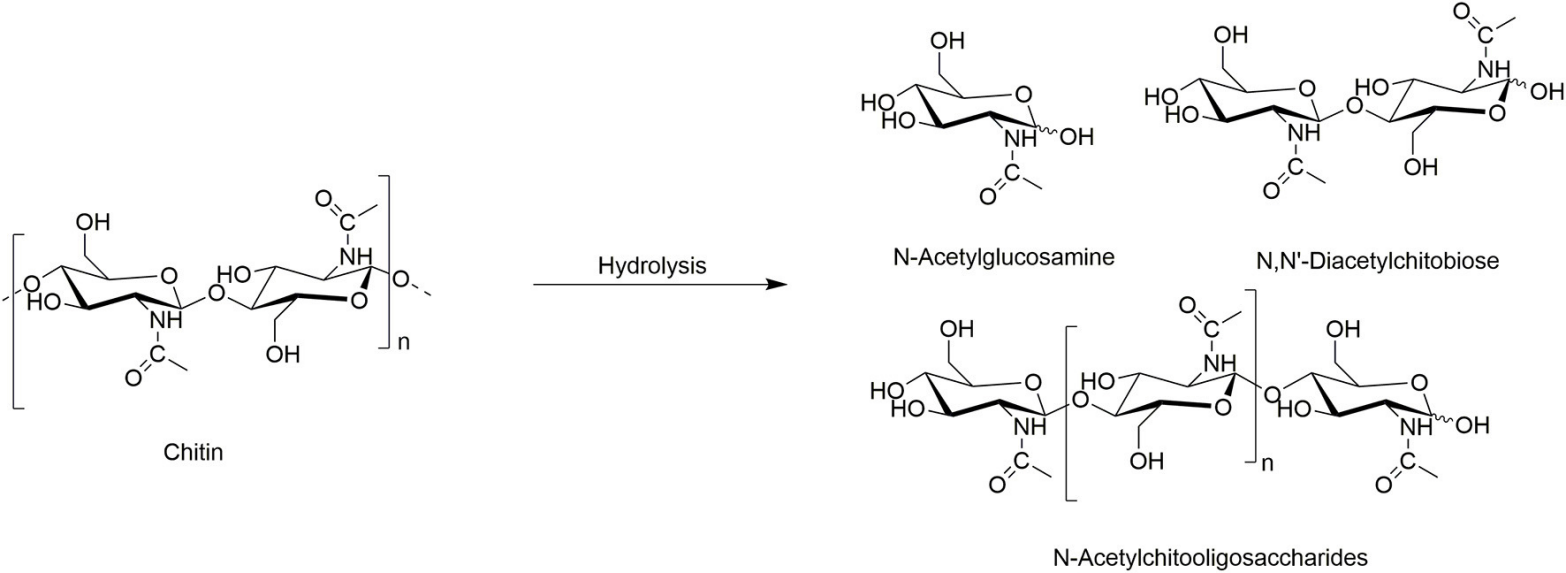
Chitin hydrolysis.

From polymers, a broad range of monomers can be obtained as a single feedstock making them a good resource for the development of renewable chemicals. Thus, the first step in chitin transformation into various chemicals is its depolymerization, a process where chitin is broken down into its basic building blocks: the monomer GlcNAc, dimer *N, N*′-diacetylchitobiose, and longer oligomers (Scheme 2). The GlcNAc is used as a food additive in the food industry (as, e.g., antimicrobial and antitumoral food additive) (Nakagawa et al., 2011), joint-pain reliever, osteoarthritis and diabetes cure (Future Market Insights, 2019), and in cosmetic (as, e.g., skin moisturizers). The dimer is known to be a plant elicitor and promote plant induced immune response (Nakagawa et al., 2011); it is also used in research of chitobiose transporter systems and enzymes (Rhodes et al., 2010).

The hydrolysis of chitin can be achieved either by enzymatic or chemical approaches. The former approach for GlcNAc production is less preferred due to its lower product yield than that in the chemical (acidic) hydrolysis. However, the highly corrosive nature of the hydrochloric acid (HCl) generally used in the process requires replacing the equipment after a certain period of time, increasing the final cost of GlcNAc (Future Market Insights, 2019).

A few examples use ILs for the hydrolysis of chitin (Table 1). The initial report used the IL 1-ethyl-3-methyl-imidazolium acetate ([C_2_mim][OAc]) to pre-treat chitin prior to enzymatic hydrolysis (Table 1, Entry 1). After pre-treatment, chitin was further hydrolyzed into the GlcNAc monomer using commercially available enzymes from *Trichoderma viride* and *S. griseus* (Husson et al., 2017). The IL-pre-treated (swollen) commercially available chitin resulted in a significant facilitation of, otherwise slow, enzymatic hydrolysis. The products of the reaction were GlcNAc (~18%), *N, N*′-diacetylchitobiose (~67%), and small amount of oligomers. The study suggested an increase in accessibility of enzymes to the chitin polymer after the IL pre-treatment.

**Table 1:** Hydrolysis and deacetylation of chitin with the use of ILs.

**S.no**	**Reaction type**	**Conditions**	**IL used**	**IL role**	**Products**	**References**
1	Enzymatic hydrolysis	*Trichoderma viride* and *Streptomyces griseus* chitinases	[C_2_mim][OAc]	Pre-treatment agent	GlcNAc, *N, N*′-diacetylchitobiose, and oligomers	Husson et al., 2017
2	Enzymatic hydrolysis	*S. griseus* chitinase	[C_2_mim][OAc]	Used to regenerate chitin using this IL	GlcNAc, *N, N*′-diacetylchitobiose, and triacetylchito-biose	Berton et al., 2018
3	Acidic hydrolysis	N/A	[HOSO_2_C_3_mim] [CF_3_SO_3_]; [HOSO_2_C_8_mim] [NTf_2_]	Solvent and catalyst	GlcNAc	Pischek et al., 2014
4	Enzymatic deacetylation	ChD enzyme from *Absidia orchidis*	[Amim]Cl; [C_4_mim]Br	Activator of ChD	Fully deacetylated chitosan	Aspras et al., 2016, 2017

The enzymatic hydrolysis of chitin was also studied using *S. griseus* chitinase but utilized different chitin substrates (Table 1, Entry 2) (Berton et al., 2018). One type of chitin was dissolved in [C_2_mim][OAc] IL and then reconstituted using water as an antisolvent remaining in amorphous, gel-like, state (amorphous wet IL-chitin). The second type of chitin was isolated with the help of [C_2_mim][OAc] IL and not otherwise pre-treated or dissolved (IL-dry-chitin). It was found that in both cases, the enzymatic reaction resulted in the formation of GlcNAc and the dimer *N, N*′-diacetylchitobiose. However, a longer chain trimer was detected in hydrolysis of regenerated amorphous wet chitin, which might be a result of its slower hydrolysis rate due to its higher hydration degree. This study pointed out the identity of obtained oligomers depending on pre-treatment of chitin with the IL and suggested that control of the hydrolysis products might be achieved using different pre-treatment conditions.

In the reactions where IL and a carbohydrate (e.g., chitin, chitosan, glucosamine, N-acetylglucosamine) is involved, the first, common step is the disruption of the hydrogen bonding of the carbohydrate, both inter- and intramolecular, and the formation of new hydrogen bonds between its hydroxyl groups and the anions of the IL. For the enzymatic hydrolysis reaction, it was shown that the enzymatic activity is strongly governed not only by the enzyme but also by the nature and amount of ionic liquid(s). For example, the enzymatic hydrolysis of chitin that has been pre-treated with [C_2_mim][OAc] (either as pretreatment solvent, a co-solvent, or both), was found to be conditioned by the structural changes of the biopolymer induced by the IL. In all cases, the IL disrupted the hydrogen bonding of the chitin molecule and increased the enzymes' accessibility to the chitin substrate. It has also been shown that simultaneous use of the IL as pretreatment solvent and co-solvent was the most efficient and required the least amount of [C_2_mim][OAc].

This was also confirmed by Li et al. (2019), who reported a significant decrease in the crystallinity of the polymer after IL pretreatment, and thus higher enzyme activity when compared to non-treated polymer. The authors hypothesized that enzymatic performances were correlated with the polymers' structural changes and depended on polymer's particle size and porosity. They evaluated N-acetylglucosamine solvation in [C_2_mim][OAc] by NMR spectroscopy studies and confirmed the disruption of H-bonding network and the formation of new H-bonds between the IL anion and chitin hydroxyl groups as the major forces for the transformation.

An interesting example of an acidic, not enzymatic hydrolysis of chitin into GlcNAc monomer was presented using acidic ILs, namely 3-(1-methyl-1H-imidazol-3-ium-3-yl)propane-1-sulfonate triflate ([HOSO_2_C_3_mim][CF_3_SO_3_]) and 8-(1-methyl-1H-imidazol-3-ium-3-yl)octane-1-sulfonate bis(trifluoromethylsulfonyl)imide ([HOSO_2_C_8_mim][NTf_2_]), that were used as both a solvent and acidic catalyst (Pischek et al., 2014) to produce GlcNAc in moderate yield (Table 1, Entry 3). Still, although a good example of the use of the IL as both the catalyst and solvent for the hydrolysis of chitin, this work was presented at a conference, and as such, there is no record of the results (yields) achieved.

## Deacetylation

**Scheme 3:**
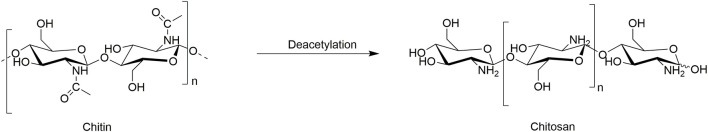
Chitin deacetylation.

The de-*N*-acetylation (or “deacetylation”) process can be used on either chitin itself (Scheme 3), chitosan, or the *N*-acetylated sugars obtained after the hydrolysis of chitin. We could find only one report where ILs were used in the enzymatic deacetylation using the enzyme chitin deacetylase (ChD) (Table 1, Entry 4). In this study, chitosan with degree of acetylation (DA) of 23% was further deacetylated using ChD enzyme isolated from *Absidia orchidis* (Aspras et al., 2016). In this reaction, the authors reported that the [C_4_mim]Br IL acts as an activator of chitin deacetylase (ChD) (Aspras et al., 2016). Since chitin deacetylase can function in an IL-free environment, this activation effect takes place via non-essential enzyme activation mechanism. The IL was proposed to bind both the enzyme and the enzyme-substrate complex, although with different binding strength. However, when the concentration of the IL becomes high, this effect decreased, possibly because of some non-specific interactions between the IL and the ChD enzyme, affecting the tertiary structure of the enzyme. Other ILs, such as composed of imidazolium and pyridinium cations (1-alkyl-3-methylimidazolium [C_2_mim]^+^, 1-allyl-3-methylimidazolium [Amim]^+^, 1-butyl-3-methylimidazolium [C_4_mim]^+^, and 1-ethylpyridinium [C_2_pyr]^+^) combined with halide (chloride Cl^−^, bromide Br^−^, iodide I^−^), bis(trifluoromethane)sulfonimide ([NTf_2_]^−^), and dicyanamide ([DCA]^−^) anions, were screened for their ability to activate ChD enzyme.

The experiments were carried out using 0.1 wt% chitosan load, 2% (v/v) ChD enzyme, and 10–70% IL in the reaction mixture. While none of the ILs acted as ChD inhibitors, [Amim]-containing ILs were able to significantly increase the activity of ChD. The highest increase in activity was noticed for [Amim]Cl IL (40% increase compared to the reaction conducted in the absence of the IL). [C_4_mim]Br IL also demonstrated a significant increase in the activity of ChD (30% increase compared to the reaction conducted in the absence of the IL), while [C_2_mim]^+^ or [C_2_pyr]^+^-derived ILs had no effect on the enzyme activity. A follow up study indicated that the activity of ChD depended on the IL concentration, and at a low concentration, [C_4_mim]Br acted as an activator for ChD. Contrarily, with an increase of IL amount, no change in enzyme activity was observed (Aspras et al., 2017).

## Production of Furan Derivatives

### Preparation of 3-Acetamido-5-Acetylfuran (3A5AF)

**Scheme 4:**
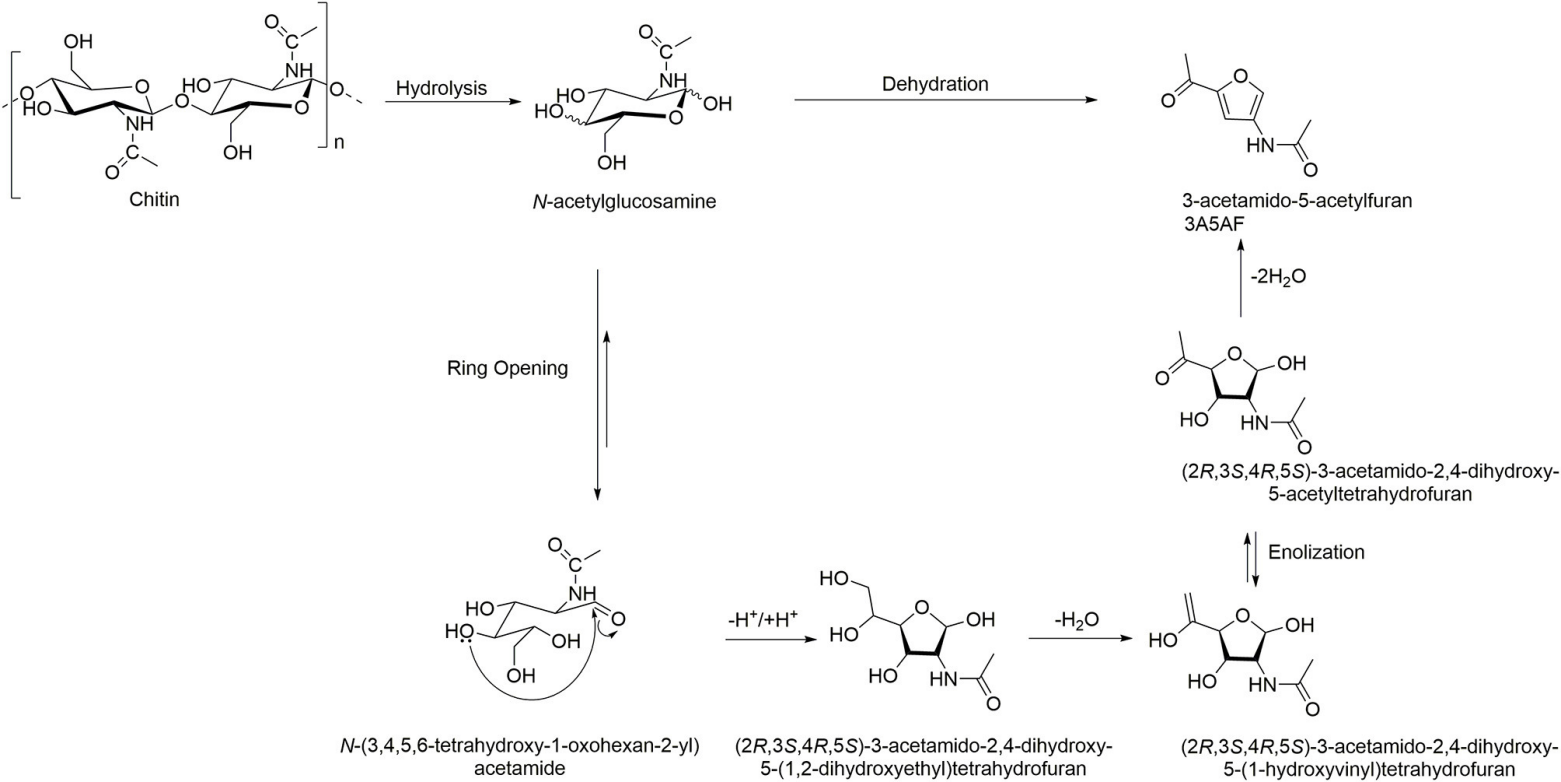
Chitin conversion into 3-acetamido-5-acetylfuran (3A5AF). Adapted from Drover et al. (2012).

One of the most studied areas of chitin transformation using ILs is its direct conversion into the furan derivative 3A5AF through hydrolysis followed by dehydration (Scheme 4, Table 2). Generally, this type of reactions is conducted under catalyzed high-temperature conditions or using microwave energy, utilizing dimethylformamide (DMF) or dimethylacetamide (DMAc) as solvents (Chen et al., 1998, 2014; Drover et al., 2012; Omari et al., 2012).

**Table 2:** Preparation of chemicals from chitin with the use of ILs^a^.

**S.no**	**Substrate**	**Conditions**	**Most successful ILs**	**IL role**	**Product (Yield, %)**	**References**
1	Commercial GlcNAc monomer	Multi-cell reactor, water/IL, 120°C, 1 h	[C_4_mim]Cl	Co-solvent	3A5AF (14.1)	Drover et al., 2012
2	Commercial GlcNAc monomer	Multi-cell reactor, water/IL, 180°C, 1 h	[C_4_mim]Cl	Co-solvent	3A5AF (25.5)	Drover et al., 2012
3	Commercial GlcNAc monomer	Multi-cell reactor, water/IL, 180°C, 1 h	[C_1_C_4_mim]Cl	Co-solvent	3A5AF (25.3)	Drover et al., 2012
4	Commercial GlcNAc monomer	Multi-cell reactor, water/IL, 180°C, B(OH)_3_, 1 h	[C_1_C_4_mim]Cl	Co-solvent	3A5AF (60.0)	Drover et al., 2012
5	Commercial chitin	Multi-cell reactor, water/IL, 8 wt% chitin load, 180°C, CrCl_3_, 1 h	[Amim]Cl	Co-solvent	3A5AF (0.5)	Chen et al., 2015b
6	Commercial chitin	Multi-cell reactor, water/IL, 8 wt% chitin load, 180°C, B(OH)_3_/HCl, 1 h	[C_2_mim]Cl	Co-solvent	3A5AF (4.5)	Chen et al., 2015b
7	Commercial chitin	Multi-cell reactor, water/IL,8 wt% chitin load, 180°C, B(OH)_3_/HCl, 1 h	[C_4_mim]Cl	Co-solvent	3A5AF (6.2)	Chen et al., 2015b
8	Commercial chitin	Multi-cell reactor, water/IL, 8 wt% chitin load, 180°C, B(OH)_3_/HCl, 10 min	[Amim]Br for dissolution-regeneration of the polymer then [C_4_mim]Cl	Co-solvent	3A5AF (28.5)	Chen et al., 2015a
9	Commercial chitin and chitosan	50 mL stainless steel vessel with a Teflon lining, sealed by a screw cap, 4 wt% IL in water, 100 mg chitosan, 180°C, 5 h	[Hmim][HSO_4_]	Co-solvent/ catalyst	5-HMF (29.5 from chitosan); (19.3 from chitin)	Li et al., 2015
10	Commercial chitin and GlcNAc monomer	50 mL stainless steel vessel with a Teflon lining, sealed by a screw cap, IL in DMSO/water, 100 mg GlcNAc, 20 of [Hmim][HSO4]/GlcNAc molar ratio, 180°C, 6 h	[Hmim][HSO_4_]	Catalyst	5-HMF (64.6 from GlcNAc); (25.7 from chitin)	Zang et al., 2017
11	Commercial chitin	Synthesis reactor with 3% w/w IL in water, Fe(ClO_4_)_3_.xH_2_O (catalyst), chitin, 6 h, 200°C	[(CH_2_)_4_SO_3_Hpy][HSO_4_]	Co-solvent	5-HMF (46)	Zang et al., 2015
12	Commercial GlcNAc·HCl	Synthesis reactor with 10 g IL, 20 g GlcNAc·HCl, H_2_O_2(aq)_, 80 mL DMSO, 10 days, 20°C	[C_2_mim][OAc]	Co-solvent/catalyst	Fructosazine (40)	Hou et al., 2015
13	Commercial GlcNAc·HCl	Synthesis reactor with 15 g IL, 35 g NaGlcN-2S, hypobromous acid, 100 mL DMSO, 10 min, 80°C	[C_4_mim][OAc]	Co-solvent/catalyst	Fructosazine (25)	Hou et al., 2015
14	Commercial chitin	Synthesis reactor with 20 g IL, 200 g chitin, KBrO, 200 mL DMSO, 5 min, 200°C	[C_2_mim][OH]	Co-solvent/catalyst	Fructosazine (15)	Hou et al., 2015
15	Commercial chitin	Synthesis reactor with 50 g IL, 45 g chitin, H_2_O_2(aq)_, 500 mL DMSO, 1 h, 180°C	[C_6_mim][OH]	Co-solvent/catalyst	Fructosazine (12)	Hou et al., 2015
16	Commercial chitosan	Synthesis reactor with 80 g IL, 60 g chitosan, H_2_O_2(aq)_, 800 mL DMSO, 9 h, 100°C	[C_4_mim]_2_[CO_3_]	Co-solvent/catalyst	Fructosazine (10)	Hou et al., 2015
17	Commercial chitosan	Synthesis reactor with 70 g IL, 15 g chitosan, H_2_O_2(aq)_, 500 mL DMSO, 6 h, 200°C	[C_4_mim][HCO_3_]	Co-solvent/catalyst	Fructosazine (28)	Hou et al., 2015
18	Commercial chitosan	Synthesis reactor with 35 g IL, 25 g chitosan, KBrO_3_, 10 mL DMSO, 5 h, 170°C	[C_4_mim][C_6_H_5_COO]	Co-solvent/catalyst	Fructosazine (54)	Hou et al., 2015
19	Commercial NaGlcN-2S	Synthesis reactor with 20 g IL, 15 g GlcNAc·HCl, H_2_O_2(aq)_, 500 mL DMSO, 5 h, 80°C	[C_2_mim][OAc]	Co-solvent/catalyst	Fructosazine (59)	Hou et al., 2015
20	Commercial GlcNAc·HCl	Synthesis reactor with 10 g IL, 1 g GlcNAc·HCl, 50 mL DMSO, 48 h, 25°C	[C_2_mim][OAc]	Co-solvent/catalyst	Deoxyfructosazine (60)	Wang et al., 2016
21	Commercial NaGlcN-2S	Synthesis reactor with 25 g IL, 50 g NaGlcN-2S, Na_2_B_4_O_7_, 10 mL DMSO, 10 min, 80°C	[C_2_mim][OAc]	Co-solvent/catalyst	Deoxyfructosazine (55)	Wang et al., 2016
22	Commercial NaGlcN-2S	Synthesis reactor with 10 g IL, 5 g NaGlcN-2S, K_2_SO_4_, 25 mL DMSO, 5 min, 150°C	[C_4_mim][OAc]	Co-solvent/catalyst	Deoxyfructosazine (65)	Wang et al., 2016
23	Commercial chitin	Synthesis reactor with 40 g IL, 35 g chitin, HOAc, 10 mL DMSO, 6 h, 150°C	[C_4_mim][OAc]	Co-solvent/catalyst	Deoxyfructosazine (15)	Wang et al., 2016
24	Commercial chitin	Synthesis reactor with 50 g IL, 45 g chitin, K_2_CO_3_, 150 mL DMSO, 4h, 180°C	[C_6_mim][OH]	Co-solvent/catalyst	Deoxyfructosazine (10)	Wang et al., 2016
25	Commercial chitosan	Synthesis reactor with 100 g IL, 60 g chitosan, H_3_PO_4_, Na_2_CO_3_, 200 mL DMSO, 9 h, 100°C	[C_4_mim]_2_[CO_3_]	Co-solvent/catalyst	Deoxyfructosazine (36)	Wang et al., 2016
26	Commercial chitosan	Synthesis reactor with 70 g IL, 15 g chitosan, KH_2_PO_4_, 10 mL DMSO, 1 h, 200°C	[C_4_mim][HCO_3_]	Co-solvent/catalyst	Deoxyfructosazine (30)	Wang et al., 2016
27	Commercial chitosan	Synthesis reactor with 35 g IL, 25 g chitosan, phenylboronic acid, 10 mL DMSO, 5 h, 170°C	[C_4_mim][C_6_H_5_COO]	Co-solvent/catalyst	Deoxyfructosazine (45)	Wang et al., 2016

a *3A5AF, 3-acetamido-5-acetylfuran; 5-HMF, 5-hydroxymethylfurfural; NaGlcN-2S, Sodium glucosamine Sulfate; Na_2_B_4_O_7_, Sodium tetraborate*.

The initial study by Kerton et al. screened six ILs for the conversion of GlcNAc monomer into 5A5AF, prior to testing chitin polymer (Table 2, Entries 1–4) (Drover et al., 2012). The ILs featured imidazolium cations and basic anions, namely [C_2_mim]Br, [C_2_mim][OAc], [C_4_mim]Br, [C_4_mim]Cl, [C_4_mim][OAc], and 1,2-dimethyl-3-butylimidazolium chloride ([C_1_C_4_mim]Cl). Out of the six tested ILs, those containing Cl^−^ as the anion showed the best performance for the transformation of the amino-sugar into the *N*-substituted furan; yields of the product were found to be 14.1 and 25.5% in [C_4_mim]Cl (at 120°C and 180°C, respectively), and 25.3% in [C_1_C_4_mim]Cl (at 180°C). Various additives were screened to increase the 3A5AF yield, and it was found that the addition of boric acid (B(OH)_3_) significantly improved the yield up to 60%.

As continuation of these studies, Kerton et al. used chitin in place of GlcNAc for the formation of 3A5AF using ILs (Table 2, Entries 5, 6) (Chen et al., 2015b). The portfolio of ILs was much broader than that in the previous study and consisted of the commercially available imidazolium ILs such as [Amim]Br, [Amim]Cl, [C_4_mim][OAc], 1-butyl-3-methylimidazolium tetrafluroborate ([C_4_mim][BF_4_]), [C_4_mim]Cl, 1-butyl-3-methylimidazolium triflate ([C_4_mim][CF_3_SO_3_]), [C_4_mim][NTf_2_], 1-butyl-3-methylimidazolium hexafluorophosphate ([C_4_mim][PF_6_]), [C_2_mim]Cl, and 1-(2-hydroxyethyl)-3-methylimidazolium chloride ([HOC_2_mim]Cl) and the in-house synthesized ILs 1-butyl-3-methylimidazolium hydrogen sulfate ([C_4_mim][HSO_4_]) and its sulfonated derivative ([HOSO_2_C_4_mim][HSO_4_]). The ILs were used with and without additives (e.g., aluminum chloride (AlCl_3_), calcium chloride (CaCl_2_), chromium chloride (CrCl_3_), cobalt chloride (CoCl_2_), nickel chloride (NiCl_2_), HCl, and B(OH)_3_).

For the screening, all reactions were conducted in a multi-cell reactor at 120–180°C using commercially available, low molecular weight (MW) chitin. It was found that conversion into 3A5AF product was successful in ILs featuring Cl^−^ anion, in which chitin had either partial or complete solubility. These ILs included [Amim]Cl, [C_2_mim]Cl, and [C_4_mim]Cl. Interestingly, no additive was required for the conversion to occur, although incorporation of an additive into the reaction significantly improved the yield. Here, 400 mol% in respect to chitin using B(OH)_3_ and 100 mol% in respect to chitin using HCl were the best performing “combinational” additives. The optimum conditions were found to be as follows: 8 wt% chitin load in [C_4_mim]Cl IL, B(OH)_3_/HCl and stirring with heating at 180°C for 1 h. These conditions resulted in a 3A5AF with 6.2% yield.

Chen et al. also reported the formation of 3A5AF from chitin, using [C_4_mim]Cl and utilizing the conditions described above, however in this case, chitin was pre-treated by various means prior to the transformation (Table 2, Entry 8) (Chen et al., 2015a). The pre-treatment included ball-mill grinding, steam, alkaline and acidic treatments, and decreasing of crystallinity through the dissolution-regeneration of the polymer from [Amim]Br IL. After pre-treatment, the procedures for chitin dehydration in IL solvent were carried out as reported by Kerton et al. (Chen et al., 2015b): 8 wt% chitin in [C_4_mim]Cl, B(OH)_3_/HCl additive at 180°C. After the reaction, 3A5AF product was extracted with ethyl acetate. The yield was improved significantly in IL solvent after ball-milling treatment, with the best yield of 28.5% being achieved within 10 min (vs. 7.5% for untreated chitin).

The first step is based on the disruption of the inter- and intramolecular hydrogen bonding of sugars and the formation of new hydrogen bonds between the carbohydrate hydroxyl protons and the anions of the IL, which facilitates the hydrolysis of chitin to its monomer. In fact, kinetic studies suggest that hydrolysis of the crystalline region of chitin is likely to be a rate-determining step (Chen et al., 2014). Then, the mechanism for formation of 3A5AF from N-acetylglucosamine is analogous to that described elsewhere for other sugars (e.g., fructose and glucose) dehydration processes. Here, complexation of imidazolium (or additive employed) with the hydroxyl oxygen of the sugar facilitates its conversion into the open chain aldose form. The nucleophilic attack by a hydroxyl group to a carbonyl yields the 5-membered heterocyclic ring, which, in turns undergoes subsequent dehydration and keto-enol tautomerization to yield the 3A5AF product.

The produced 3A5AF can be further employed as a building block in a multistep synthesis. New applications and markets for this chemical include its incorporation into active pharmaceutical ingredients (APIs), such as proximicins, aminofuran antibiotics, and anticancer compounds isolated from marine strains MG-37 of the actinomycete *Verrucosispora* (Fiedler et al., 2008). Sadiq et al. developed a safe, sustainable synthesis of proximicin A (Sadiq et al., 2018) from 3A5AF prepared from mechanochemically treated chitin by Chen et al. (2015a) (Table 2, Entry 7), i.e., by using [C_4_mim]Cl as a solvent, and B(OH)_3_/HCl additives. The reaction sequence from 3A5AF proceeded through 6 steps, namely the oxidation of 3A5AF into the corresponding ester, selective hydrolysis of respective amide, installation of the methyl carbamate moiety, selective ester hydrolysis, and finally selective formation of amide using ammonium hydroxide coupling with 3-aminofuran. Although an IL was used to generate 3A5AF from chitin, their use in the following steps is still to be explored. Still, the proposed synthesis eliminated the use of toxic chemicals previously employed for this transformation. In addition, the study emphasized the role chitin would be playing in the sustainable production of nitrogen-containing building blocks, which are not directly obtainable from lignocellulosic biomass.

### Preparation of 5-Hydroxymethylfurfural (5-HMF)

**Scheme 5:**
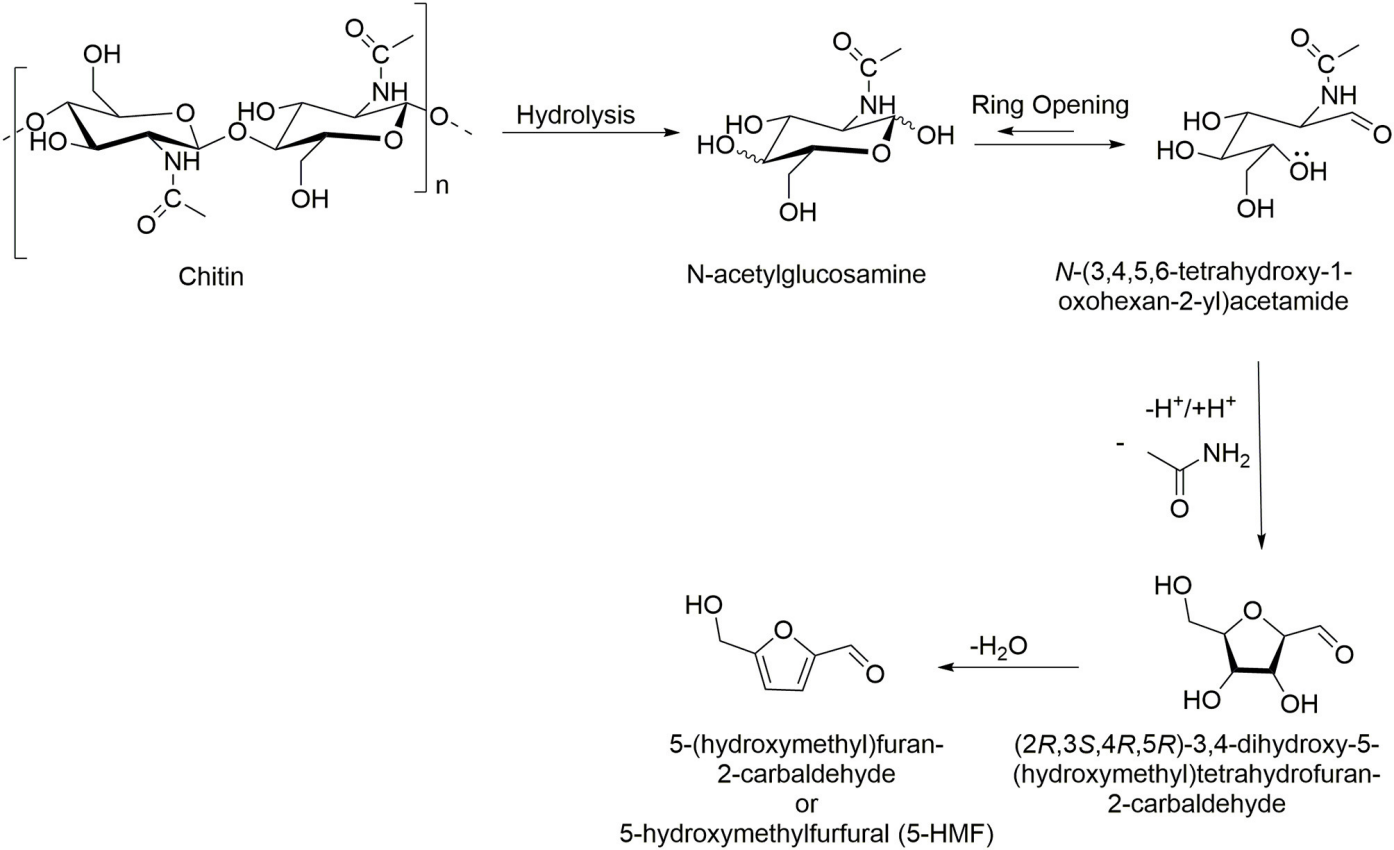
Chitin conversion into 5-hydroxymethylfurfural (5-HMF).

The direct conversion of chitin into 5-HMF is a traditional reaction usually conducted using metal salts as catalysts (FeCl_2_, ZnCl_2_, AlCl_3_, and B(OH)_3_) under high temperature, hydrothermal conditions (Scheme 5) (Wang et al., 2013; Yu et al., 2016). In 2015, ILs with different cations and anions of varying acidity, hydrogen-bonding capacity, and steric hindrance were tested as catalysts for 5-HMF formation from chitosan as the starting material (Table 2, Entry 9). The ILs included [C_4_mim][HSO_4_], [C_4_mim][BF_4_], [C_4_mim]Cl, [C_4_mim]Br, 1,3-bis(4-sulfobutyl)-1H-imidazol-3-ium hydrogen sulfate [(HOSO_2_C_4_)_2_im][HSO_4_], 3-methyl-1-(4-sulfobutyl)-1H-imidazol-3-ium hydrogen sulfate [HOSO_2_C_4_mim][HSO_4_], 1-methylimidazolium hydrogen sulfate ([Hmim][HSO_4_]), 1-methylimidazolium chloride ([Hmim]Cl), and 1-carboxymethyl-3-methylimidazolium hydrogen sulfate ([HOOCCH_2_mim][HSO_4_]) (Li et al., 2015).

The nine ILs were initially evaluated using chitosan as a substrate, five of which performed satisfactorily, although the reaction required a high temperature (180°C). The best performances (based on yields) were [Hmim][HSO_4_] (21.7% yield at 2 wt% IL loading and 29.5% yield at 4 wt% IL loading) > [C_4_mim][HSO_4_] (18.9% yield) > [(HOSO_2_C_4_)_2_im][HSO_4_] (12.8% yield) > [HOOCCH_2_mim][HSO_4_]) (11.5 % yield) > [C_4_mim][BF_4_] (10.8% yield). After using chitosan for the reaction optimization, the best performing IL, [Hmim][HSO_4_], was tested on chitin under the optimized conditions (0.5 wt% chitin suspension in water, 5 h treatment time, 4 wt% [Hmim][HSO_4_] IL, at 180°C) resulting in 19% yield of HMF. As a comparison, the reaction in the absence of any catalyst did not form any product, and the yield of the reaction that used SnCl_4_·5H_2_O or ZnCl_2_ resulted in only 10% yield of HMF.

A series of Brønsted acidic ILs with different structures were also tested for the conversion of chitin and its derivative into 5-HMF (Table 2, Entry 10). The ILs included [Hmim][HSO_4_], 1-methylimidazolium dihydrogen phosphate ([Hmim][H_2_PO_4_]), 1-methylimidazolium nitrate ([Hmim][NO_3_]), [Hmim]Cl, 4,5-dimethylthiazolium hydrogen sulfate ([TM][HSO_4_]), 2-isobutylthiazolium hydrogen sulfate ([TB][HSO_4_]), and 4,5-dimethyl-3-(4-sulfonic acid butyl)thiazolium hydrogen sulfate ([TBSO_3_H][HSO_4_]). Initially the reaction was conducted using GlcNAc as a substrate, and then was extended to the use of chitin and chitosan under the best determined conditions.

With exception of [Hmim][HSO_4_], the other ILs resulted in a moderate conversion of GlcNAc into 5-HMF (traces to 28.7% yield). The IL [Hmim][HSO_4_] exhibited the best catalyst performance (45.2% yield) which, when optimized, reached 64.6% yield without the need of catalysts other than the IL. The reaction was conducted using GlcNAc/water/dimethylsulfoxide (DMSO) ratio of 1/120/80 by weight, under 180°C for 6 h. When chitin was employed as a substrate, the usage of [Hmim][HSO_4_] IL resulted in 25.7% yield of HMF under these conditions, providing access to valuable chemicals from chitin for biorefinery purposes.

A patent application (Zang et al., 2015) has also used chitinous raw materials for their catalytic conversion into 5-HMF, employing ILs as co-solvents and catalysts. Both chitin and chitosan were studied: several chotosans with DA in the range of 20–50% and several chitins with DA in the range of 85–95%. Some of the ILs used in this study were the same as previously noted, namely [C_4_mim]Cl, [C_4_mim][OAc], [Amim][OAc], [Hmim][HSO_4_], but also included novel ILs (not previously evaluated for this application): 3-methyl-1-(4-sulfobutyl)-1H-imidazol-3-ium hydrogen sulfate [HO_3_S(CH_2_)_4_mim][HSO_4_], lysine 4-methylbenzenesulfonate [Lys][pCH_3_C_6_H_4_SO_3_], 1-carboxy-3-methylimidazolium chloride [HOOCmim]Cl, glycine chloride [Gly]Cl, 1-(4-sulfobutyl)pyridinium hydrogen sulfate [HO_3_S(CH_2_)_4_Py][HSO_4_]. The reaction was conducted in water as a solvent, with 0.25–0.33 wt% load of chitin or chitosan to form a suspension and 1–20 wt% IL as a co-solvent and acidic catalyst. This reaction was conducted with or without additional catalysts (trace amounts of La(CF_3_SO_3_)_3_, CrCl_3_·6H_2_O, Gd(CF_3_SO_3_)_3_·H_2_O, Yb(CF_3_SO_3_)_3_·H_2_O, etc.), under 120–200°C heating. Unfortunately, due to the patent being relatively broad, it is not clear which ILs were the best performing. The best reported yield of 5-HMF (46%) was achieved using [(CH_2_)_4_SO_3_Hpy][HSO_4_] as co-solvent and Fe(ClO_4_)_3_·H_2_O as catalyst.

Similarly for 5-HMF, the first step of the reaction no matter whether it is done from chitin or chitosan is the formation of new hydrogen bonds though the coordination of basic anions with chitin's or chitosan's hydroxyls, which breaks the original hydrogen bonding (This step is similar to all related chitin biorefinery reactions.). Then, the mechanism is very similar to that for formation of 3A5AF from *N*-acetylglucosamine, followed by the released of H^+^ and/or electron-rich aromatic, which facilitates the conversion of the carbohydrate into its open chain aldose form; this is the rate-limiting step. This step is followed by the isomerization into enol-intermediate, followed by ring closure with simultaneous deamination. Subsequent keto-enol tautomerization into 4-hydroxy-5-hydroxymethyl-4,5-dihydrofuran-2-carbaldehyde followed by dehydration yields the desired 5-HMF product (Li et al., 2015).

## Preparation of Complex Compounds

The preparation of fructosazine [IUPAC name: 2,5-bis(1,2,3,4-tetrahydroxybutyl)pyrazine]) and deoxyfructosazine (Scheme 1) from chitin was also reported (Hou et al., 2015; Wang et al., 2016). These compounds are used as flavoring agents, fragrance compounds, and in medicine for the prevention and treatment of osteoarthritis and as popular anti-inflammatory agents (Leffingwell, 1976; Giordani et al., 2006; Yang and Yan, 2018).

To produce fructosazine (Table 2, Entries 12–19), chitin biomass was oxidized with strong oxidizer in DMSO, with an IL catalyst which also played a co-solvent role; traditionally this reaction requires inorganic salts as catalysts (Hou et al., 2015). The ILs evaluated were the imidazolium ILs [C_2_mim][OAc], [C_4_mim][OAc], 1-ethyl-3-methylimidazolium hydroxide ([C_2_mim][OH]), 1-butyl-3-methylimidazolium hydroxide ([C_4_mim][OH]), 1-butyl-3-methylimidazolium carbonate ([C_4_mim]_2_[CO_3_]), 1-butyl-3-methylimidazolium bicarbonate ([C_4_mim][HCO_3_]), and 1-butyl-3-methylimidazolium benzoate ([C_4_mim][C_6_H_5_COO]). The chitin raw material was loaded into DMSO/IL mixture (DMSO to chitin ratio of 10:1 w/w, IL to chitin ratio of 1:2–100 w/w), and then oxidant added (oxidant to chitin ratio of 1–50:1 w/w). Aqueous hydrogen peroxide, sodium hypochlorite, sodium hypobromite, or potassium hypobromite (30–95% solutions in water) were used as oxidants. The reactants were mixed and then allowed to react at temperatures up to 200°C, for up to 10 days. The product was easily recrystallized from the reaction mixture using acetonitrile, ethanol, propanol, or acetone as recrystallization solvents. Unfortunately, details or more narrow ratios between reagents were not provided as the patent is broad.

Deoxyfructosazine was prepared using very similar conditions as those reported above (Table 2, Entries 20–27): DMSO was used as a solvent, ILs as co-solvent/catalyst, and a dehydration agent (B(OH)_3_, sodium tetraborate, phenylboronic acid, acetic acid, AcOH, carbonic acid or phosphoric acid) (Wang et al., 2016). While the reaction was also attempted in DMF and DMAc, the use of DMSO resulted in the highest product yield. The reaction was carried out in a pressure vessel with a Teflon screw cap. Chitin, DMSO, IL and an additive were placed in a pressure vessel equipped with a magnetic stirring bar, and a reaction was heated in an oil bath (25–200°C) at different time intervals (from 5 min to 2 days). While the ratios of reagents depended on the IL identity and the dehydration agent used in the reaction, in general the ratio of IL:chitin was ca. 1–1.5:1 w/w, 2–50:1 mol/mol dehydrating additive:chitin, IL/DMSO 0.25:1–50 v/v.

Using low MW chitin (MW 250,000 Da), [C_4_mim][OAc] IL was employed as catalyst, AcOH as dehydration agent, and the ratio of reagents was 1.2:1 w/w IL:chitin, 2:1 mol/mol AcOH:chitin, 4:1 v/v IL:DMSO, at 150°C for 6 h. In another example, [C_6_mim][OH] IL and K_2_CO_3_ as a dehydration agent were utilized for conversion of high MW chitin (MW 1,000,000 Da). The ratio of reagents was 1.1:1 w/w IL:chitin, 15:1 mol/mol K_2_CO_3_:chitin, 1:3 w/w IL:DMSO, at 180°C for 4 h. The yield of deoxyfructosazine was determined to be 50–60%. Interestingly, the use of ILs with acidic sulfonic group that are usually used as dehydration agents in similar reactions as catalysts required increase in the reaction temperature and resulted in bond cleavage with formation of formic and levulinic acids, and not deoxyfructosazine (Wang et al., 2016). Recently, selective transformation of chitin and chitosan into levulinic acid has been realized by the catalysis using acidic 1-methyl-3-(3-sulfopropyl)imidazolium hydrogen sulfate ([HSO_3_C_3_mim][HSO_4_]) up to a yield of 67.0% (Hou et al., 2019). The authors suggested the IL was not basic enough to completely disrupt H-bonding of the *N*-acetyl groups which shielded the accessibility of glycosidic linkages to the acidic catalyst, thus deacetylation-depolymerization mechanisms occurred only at the outer surface of the polymer.

## The Role of Ionic Liquids

Although enzymatic reactions (hydrolysis and deacetylation) could proceed without use of IL (IL is considered to be non-essential activator), ILs disrupt the H-bonding and thus facilitate the access of the enzyme to the substrate. This results in formation of different products and different product distribution profile (ratio of monomer to dimer to oligomers) than what is observed without the use of ILs (Husson et al., 2017; Berton et al., 2018). The IL systems also appear to be superior in terms of product yields and reaction speed.

In those cases reporting the formation of small molecule heterocycles (5-HMF, 3A5AF), no product could be formed from chitin using VOCs instead of ILs, even at harsh reaction conditions. Besides, in many cases, the product was not stable at high temperatures or for elongated reaction times, while the use of ILs allows mild reaction conditions (Chen et al., 2015b). For example, no 3A5AF was formed by using organic solvents even at a high temperature (Chen et al., 2015b). One of the reasons for this phenomenon is that there is a strong correlation between the hydrogen-bonding network strength and the reactivity of chitin, and ILs allow an easy disruption of this H-bond network (Chen et al., 2015a).

The pretreatment of chitin may be an essential step in all reactions for chitin deconstruction because it allows faster chitin depolymerization through breaking down the polymer's robust structure and H-bonding network. Considering this, ILs that possess anions that are more basic should be investigated more closely. Rational design directed to functionalization of ILs that could assist in this step may be necessary, in order to improve the reaction yield from chitin, which currently remain low.

## Recovering of Ionic Liquids

Contrarily to methods for IL dewatering (Abu-Eishah, 2011; Kuzmina and Hallett, 2016; Zhou et al., 2018), the literature addressing the recyclability of the ILs after deconstruction of chitin polymers into chemicals is scarce. The process of IL recycle would not be as simple as a dewatering of the ILs after formation of chitin *materials*, but instead we envision a two-step process, with the recovery of products and unreacted starting materials first, and then the IL purification and recovery. We will review the methods that were reported by authors of these technologies where available.

The simplest IL recovery strategy would potentially be after enzymatic hydrolysis reaction, where IL is used for the polymer's pretreatment and then is washed out with water prior to the reaction of the sugar with enzymes. Such “pre-treatment” strategy facilitates purification of mono-/oligosaccharides from the reaction media. At the same time, washing chitin out with water not only leads to a production of hydrolysates, which are nearly free of residual ILs, but also results in an IL that is simply “wet.” A simple dewatering step could be done to recover of the IL for the next pretreatment cycle. The dewatering strategies for the IL are provided elsewhere (Shamshina, 2019).

In the reactions where small molecule heterocycles (3A5AF or 5-HMF) are formed, the product is normally extracted with a suitable volatile organic solvent (VOC) such as ethyl acetate, diethyl ether, or dichloromethane, after which the IL is recovered together with the additive or catalyst used (Chen et al., 2015a). In this recovery process, the water is removed through evaporation under high-vacuum, usually using a rotary evaporator. The IL/additive that was used as catalyst remained unseparated and are used “as is,” in the next catalytic cycle. For instance, in the formation of 5HMF from glucosamine (Li et al., 2019), the recyclability of [Hmim][HSO_4_] catalyst was evaluated over five cycles on the “model” reaction, in a DMSO/water mixture. In every cycle, IL/catalyst was recovered, re-used, and the product yield was evaluated. The product yield slightly decreased (*ca*. ~10% of initial yield) with each re-use cycle, which was attributed to the catalyst mass loss during the recycle procedure. It was found that unreacted starting material and 5-HMF product can be easily removed from the reaction mixture by extraction with ethyl acetate. After removal of the product, the water was evaporated under vacuum and the remaining [Hmim][HSO_4_]/DMSO used directly in the next catalytic cycle, under the same reaction conditions.

Although not reported in the literature reviewed for chitin conversion, there are two methods that might be of interest and that would not depend of the components of the mixture to be separated. The first method is the recovery of the IL through the formation of the distillable carbene, patented by BASF (Earle and Seddon, 2001). The method involves the treatment of imidazolium IL with a strong base (such as potassium *tert*-butoxide), which deprotonates the imidazolium cation at C-2 position, forming 1,3-dimethyl-imidazol-2-ylidene carbene. This carbene is distillable and could be distilled out (Earle and Seddon, 2001). Afterwards, the formed carbene reacts with the acid of the desired anion, reforming the imidazolium IL. The method can be used after the desired products have been extracted from the IL, and the IL/residues mixture has been dewatered fully.

Membrane separation might constitute another method for the IL recovery, including the commercially available pervaporation systems (PVs). These systems have been shown to be suitable for the recycling of [C_2_mim][Ac] IL after lignocellulosic biomass pretreatment. The inventors of the technology claim separation factors of 1,500 and recycle efficiency of >99.9 wt% IL (Campos et al., 2014). This said, the efficiencies of removing contaminants by membrane separation processes would be dependent on the size and molecular weight of the mixture constituents. Either way, we are convinced that the choice and application of the separation method would depend on the product yield, other contaminants (e.g., starting materials) level, cost of the process, and chemical nature of components.

## Outlook

Every so often in the history of society, a paradigm shift, an important change that happens when the traditional way of thinking is replaced by a new and/or different way, takes place. The reason behind such change is that “crisis simultaneously loosens the stereotypes and provides the incremental data necessary for a fundamental paradigm shift” (Kuhn, 1962). Nowadays, it is all about a shift from oil-based economy to biopolymers-based one, caused by depletion of oil resources, and environmental awareness.

Due to numerous worldwide-enforced legislative measures around environmental protection, and public environmental awareness, biopolymers have finally started gaining recognition in multiple industrial sectors. Such replacement of oil-based chemicals with biopolymer-derived ones, should, however, be done in a clever fashion and careful decisions must be made whether the biopolymers should be used in their unmodified state for high-value products, or in a bio-/chemical transformation for low-value commodity chemicals. For chitin, IL-extracted chitin would be considered a specialty polymer, due to its unprecedented high molecular weight, while pulped chitin would be considered a bulk commodity. In this case, prices for the polymer would differ greatly reflecting not only more expensive process of isolation for IL-extracted chitin, but also development costs for its special molecular weight.

Since market for chemicals made from renewable biopolymers such as cellulose or chitin is driven by a low tech, but high-volume commodity, chitin obtained by pulping (Shamshina et al., 2016) would work as a suitable starting material for chitin conversion into chemicals, and provide access to the chemicals that are cannot be obtained otherwise. It is cheap and commercially available, and thus would be ideal for the purpose (Shamshina et al., 2019). A large number of existing National programs is indeed built around such bio-refineries where biomass feedstock, after partial or complete separation into main fractions, is followed by the deconstruction of the components into valuable chemicals. This includes the program by the National Renewable Energy Laboratory (NREL, 2019), and the program by Joint BioEnergy Institute (JBEI, 2019)^1^ which focus on the production of bio-based chemicals. On the other side, we believe in taking advantage of polymers accessible directly from Nature as primary polymer source for the preparation of materials, and not chopping them up into the chemicals. Here, high-cost, high-value medical devices, and implantables of IL-extracted chitin would more than cover the cost of its isolation and other chitin-based products will become more financially attractive.

Review of scientific literature suggests that biopolymers in general, and chitin in particular, are very appealing for overall biorefinery, although further research is needed for proper biopolymers' valorization. Yet, the principle of circular economy encourages its exploitation as an alternative resource for a sustainable future.

## Author Contributions

JS and PB together created the idea, the first draft, finalized, and templated the article.

### Conflict of Interest

JS is a former CSO of Mari Signum Mid-Atlantic, LLC. The remaining author declares that the research was conducted in the absence of any commercial or financial relationships that could be construed as a potential conflict of interest.

## Footnotes

^1^JBEI. The Joint BioEnergy Institute (JBEI): U.S. Department of Energy (DOE) Bioenergy Research Center. Available online at: https://www.jbei.org/about/ (accessed June 25, 2019).
